# Pharmacogenetics in Psychiatry: An Update on Clinical Usability

**DOI:** 10.3389/fphar.2020.575540

**Published:** 2020-09-11

**Authors:** Ron H. N. van Schaik, Daniel J. Müller, Alessandro Serretti, Magnus Ingelman-Sundberg

**Affiliations:** ^1^ Department of Clinical Chemistry, Erasmus MC, University Medical Center, Rotterdam, Netherlands; ^2^ Pharmacogenetics Research Clinic, Campbell Family Mental Health Research Institute, Centre for Addiction and Mental Health, Toronto, ON, Canada; ^3^ Department of Psychiatry, University of Toronto, Toronto, ON, Canada; ^4^ Department of Biomedical and NeuroMotor Sciences, University of Bologna, Bologna, Italy; ^5^ Pharmacogenetics Section, Department of Physiology and Pharmacology, Karolinska Institutet, Stockholm, Sweden

**Keywords:** pharmacogenetics, cytochrome P450, CYP2D6, CYP2C19, depression, psychiatry, antidepressant

## Abstract

Using pharmacogenetics in guiding drug therapy experiences a steady increase in uptake, although still leads to discussions as to its clinical use. Psychiatry constitutes a field where pharmacogenomic testing might help in guiding drug therapy. To address current challenges, this minireview provides an update regarding genotyping (SNP analysis/arrays/NGS), structural variant detection (star-alleles/CNVs/hybrid alleles), genotype-to-phenotype translations, cost-effectiveness, and actionability of results (FDA/CPIC/PharmGKB) regarding clinical importance of pre-emptive pharmacogenomic testing for prescription of antidepressants and antipsychotics.

## Introduction

Regarding DNA testing for guiding drug therapy, the value of *CYP2D6* and *CYP2C19* genotyping for optimizing drug treatment in psychiatry has been a focus point. Mental illness is a major health issues and has great individual and social-economical impact. In 2010, costs of mental disorders in US were USD$ 2.5 billion, and these are expected to increase considerably (Corponi et al., 2018). Rate of response to initial antidepressant treatment was only 49.6% (STAR*D trial (Rush et al., 2006)), and a systematic review showed that non-responders to one or more treatments have a 15% likelihood of suicide ideation compared to 6% of patients with treatment-responsive depression and 1% in the general population (Mrazek et al., 2014). Costs for managing nonresponders are USD$10,000/year/patient higher as compared to responsive patients (Mrazek et al., 2014). Currently, >200 drugs are available for treatment of psychiatric/neurologic patients (Hiemke et al., 2018). Use of this medication is hampered by side-effects and lack of effectivity, leaving therapeutic outcomes nonsatisfactory. Only 30% of patients suffering from major depressive disorder (MDD), bipolar disorder (BD), and schizophrenia remain compliant with medication and reach full and stable remission (Corponi et al., 2018), whereas 30–50% of patients with MDD do not respond to their first antidepressant (Rush et al., 2006). Remission rates for SSRI’s are as low as 37% (Thase et al., 2010). Regarding side effects, 25,000 patients in US present to the emergency department each year due to antidepressant-induced adverse events (Hampton et al., 2014). A major determinant affecting side effects and lack of efficacy is the relation between dosage and systemic exposure to the drug. Therapeutic drug monitoring can be used to guide antidepressant therapy. Most antidepressants/antipsychotics are being metabolized by CYP2D6, CYP2C19, and CYP3A4 enzymes in the liver (Hiemke et al., 2018). Because of the strong relation between genetic variants and enzymatic activity, analysis of *CYP2D6* and *CYP2C19* has been an early focus for the clinical use of pharmacogenetics in psychiatry. A summary of the relation between *CYP2D6*/*CYP2C19* genotypes and adjusted dose was published in 2013 (Stingl et al., 2013). Several evidence-based dosing guidelines for using pharmacogenetics for antidepressants/antipsychotics have been published (Swen et al., 2008; Swen et al., 2011; Hicks et al., 2013; Hicks et al., 2017). This mini-review addresses the latest developments in pharmacogenetics for psychiatry and discusses some challenges to be faced in the near future.

## Prospective Randomized Controlled Clinical Trials for Antidepressants

The relation between genotype and enzymatic activity is undisputed, as is the relationship between genotype and plasma concentration of a drug upon a specific dose. Yet, a major argument hampering clinical guidelines is evidence for improving clinical outcome. One of the first studies addressing clinical benefit of using pharmacogenetic information to guide drug therapy was published by Hall-Flavin et al. in 2013, showing a significant increase in responders after 8 weeks of antidepressant therapy when genetic information on *CYP2D6*, *CYP2C19*, *CYP1A2*, *SLC6A4*, and *HTR2A* was used to dose patients (n = 114) as compared to standard treatment (n = 113) (Hall-Flavin et al., 2013). In a recent meta-analysis, taking into account five prospective randomized-controlled trials on depressive symptom remission, published between 2013 and 2019 (Winner et al., 2013; Singh, 2015; Perez et al., 2017; Bradley et al., 2018; Greden et al., 2019), these initial findings were confirmed: in a total of 1,737 subjects, patients receiving pharmacogenetic-guided therapy (n = 887) were 1.71 times more likely to achieve symptom remission as compared to patients receiving usual treatment (p = 0.005) (Bousman C. A. et al., 2019). In a study on 2,066 patients, the CYP2C19 UMs and CYP2C19 PMs were more prone to switch escitalopram to another drug (Jukic et al., 2018). These studies indicate additional value of using genetics in guiding antidepressant therapy. However, it is important to realize that also negative results have been published. An excellent overview of positive and negative studies is summarized in a recent systematic review by Solomon et al. (2019), analyzing 16 studies published between 2013 and 2018. Some explanations mentioned for lack of positive associations were: non-randomization and underpowered studies, time of measuring the investigated endpoint, concomitant use of herbal remedies, unjustified exclusion of patients from the study, focus on particular ethnic groups, or more complex pharmacokinetics in relation to clinical outcome (i.e., venlafaxine metabolism).

## Retrospective, Confirmatory Cohort-Study for Antipsychotics

A recent study on aripiprazole and risperidone (both CYP2D6 substrates) using data from 2005 to 2018 from Diakonhjemmet Hospital, Oslo, Norway, showed that, without prior knowledge of *CYP2D6* genotype at the time of treatment, clinicians reduced the daily risperidone dose for CYP2D6 poor metabolizers by an average of 19% (95% CI, 5–35; p = 0.010) and for aripiprazole by 15% (95% CI, 1–28; p = 0.033). The estimated dose reduction based on pharmacogenetic constitution of the patients would have been 40 and 35%, respectively (Jukic et al., 2019). The large number of patients (725 risperidone-treated and 890 aripiprazole-treated patients) makes this one of the larger studies in the field. The incidence of switching of risperidone to another antipsychotic was significantly higher in CYP2D6 ultra-rapid metabolizers (OR, 2.9; 95% CI, 1.4–6.0; p = 0.003) and for CYP2D6 poor metabolizers (OR, 1.9; 95% CI, 1.1–3.1; p = 0.015), indicating that, at least for risperidone, *CYP2D6* genotype status has a clinical impact.

## FDA, CPIC, PharmGKB, and DPWG

Translating published evidence on pharmacogenetics into clinical actions is an important aspect needed for successful implementation. Both the Dutch Pharmacogenetics Working Group (DPWG; started in 2005) and the Clinical Pharmacogenetics Implementation Consortium (CPIC; started in 2009) use thorough review of the literature by a combination of experts in a transparent way. DPWG now has dosing advice for 94 drugs^1^ and CPIC for 54 drugs^2^. DPWG published evidence-based dosing recommendations on *CYP2D6* and *CYP2C19* genotypes for antidepressants and antipsychotics in 2008, with an update in 2011 (Swen et al., 2008; Swen et al., 2011). These recommendations are currently used in the Netherlands by all pharmacists to advice patients and physicians on drug choice and drug dosing. The Clinical Pharmacogenetic Implementation Consortium (CPIC^3^) published guidance for using genetic information in drug therapy for Psychiatry (Hicks et al., 2013; Leckband et al., 2013; Hicks et al., 2015; Hicks et al., 2017; Phillips et al., 2018; Brown et al., 2019). Information on enzymes involved in drug metabolism is also present in the drug label of more than 160 drugs^4^, but usually no specific dose recommendations are included. It must, however, be emphasized that recommendations on dosing based on genotype are not always the same between the different expert groups, and for some drugs to a great extent disparate from each other. In addition, the implementation of pharmacogenetic information into the product characteristic (SmPCs) is only found in about 50% of cases (Ingelman-Sundberg, 2020).

The PharmGKB website hosts a huge amount of relevant information on pharmacogenetics, with at present 753 drug label annotations, 154 clinical guideline annotations, 149 curated pathways, and 700 annotated drugs. In 2018, the FDA issued a safety communication indicating a lack of clinical evidence supporting the utility of pharmacogenetic testing, specifically addressing the use of pharmacogenetics for antidepressants. This letter highlighted difference in opinion exist when judging published evidence, as stated in the recent perspective on antidepressant pharmacotherapy (Hicks et al., 2020). To create clarity, FDA published in 2020 a Table of Pharmacogenetic Associations^5^, distinguishing three different categories: (a) pharmacogenetic associations for which data support therapeutic management recommendations, (b) pharmacogenetic associations for which data indicate a potential impact on safety or response, and c) pharmacogenetic associations for which data demonstrate a potential impact on pharmacokinetic properties only. Comparing this list with CPIC guidelines and DPWG recommendations (**Supplementary Table 1**), not all drugs are present in the FDA listing. Also, it can be seen in this table for which gene/drugs pairs there is agreement in proposed action, and where there is a difference in opinion. Although the FDA table is helpful in distinguishing which drugs could benefit from a pharmacogenetic test, it also shows difficulties in reaching a uniform guidance, even within the FDA. The FDA-statement from 2018 that “the relationship between DNA variations and the effectiveness of antidepressant medication has never been established”^6^ seems to be a direct contradiction of the randomized-controlled clinical trials on antidepressants mentioned earlier in this paper. Also, the note from the FDA that “the relationship between *CYP2C19* genotype and drug response to escitalopram and sertraline is not established, and this relationship is not described in the FDA-approved labelling of the drug” seems to conflict with the FDA product label for escitalopram that mentions that “the exposure under supratherapeutic 30-mg dose is similar to the steady-state concentrations expected in CYP2C19 poor metabolizers following a therapeutic dose of 20 mg”, as pointed out by Hicks et al. (2020). Also, for sertraline, which is not mentioned in the FDA table, there is substantial scientific evidence that indicates that CYP2C19 poor metabolizers have an approximately three-fold higher exposure to the drug as compared to normal metabolizers (Hicks et al., 2020). Indeed, plasma levels of antidepressants are associated with clinical outcome (Florio et al., 2017; De Donatis et al., 2019) and genetic pharmacokinetic variants showed a clinically relevant effect (Fabbri et al., 2018). Both CPIC and DPWG have adjusted dosing recommendations based on literature for sertraline and CYP2C19 PMs (**Supplementary Table 1**). Also interesting is that for tetrabenazine, for which genetic testing is required according to FDA, neither a PharmGKB clinical annotation nor a CPIC or DPWG guideline is available. It will be clear that the clinical field would benefit from clinical decision support tools, such as, for example, GeneSight, Translational Software, Corriel, PillCheck, OneOme, and Abomics. However, it should be clear which guidelines and which interpretations these dose recommendations originate from, to avoid conflicts in dosing advice. Again, harmonization would greatly help the field.

## Cost Effectiveness of Pharmacogenetic Testing

An important aspect of using pharmacogenetics, besides helping patients to reach therapeutic drug concentrations more quickly, are costs associated with this approach. One of the challenges is that pricing of health care costs as well as pharmacogenetic testing will differ between laboratories and across countries. This causes conflicting reports, as discussed by Rosenblat et al. (2017) and Peterson et al. (2017). A recent paper of Maciel et al. (2018), addressing cost savings of pharmacogenetic testing for depression in a real-world clinical setting, calculated a saving of USD$3,962 annually per patient, assuming a test cost of USD $ 2,000 (NeuroID genetix panel with 10 genes). Hornberger et al. (2015) calculated savings of USD $3,647 per patient using a USD $2,000 PGx testing panel. For comparison, cost of *CYP2D6*/*CYP2C19* genotyping in The Netherlands is between €100 and €300, thus much lower, strongly reducing expenses as compared to the US study. The impression is thus that indeed pharmacogenetic testing may be highly beneficial, also from an economical point of view. Developing countries can benefit from the knowledge obtained from developed countries, and in such implement pharmacogenetics into their healthcare system thus preventing adverse drug reactions and associated costs but also because a once in a lifetime genetic test can be more easily performed as compared to measuring drug concentrations. An approach to consider would be *CYP2B6* genotyping for efavirenz therapy, in the battle against HIV. Yet, costs, logistics, and knowledge about specific variants occurring in these countries are challenges to be addressed. The potential for implementation of pharmacogenetics in developing countries is reflected upon in several publications (Mitropoulos et al., 2011; Roederer et al., 2011; Mizzi et al., 2016; Mitropoulos et al., 2017).

In general, a more cost-effective approach might be to have for each patient a DNA passport for medication, covering most polymorphic genes involved in commonly prescribes drugs, and for which dosing recommendations are available. This would increase the benefit of pharmacogenetic tests, also beyond psychiatry, and would avoid that each separate clinical field would have to worry about cost effectiveness. In fact, the large European trial Ubiquitous PGx^7^ is investigating this approach, monitoring both medical benefits and cost-effectiveness. The outcome of this study is expected in 2020/2021.

## Genotyping Challenges

In laboratory settings, it is advocated that only tests are performed that are clinically actionable. For psychiatry, this holds true for *CYP2D6* and *CYP2C19*. The genotyping field has identified which variants per gene should be investigated, since the reliability of the predicted phenotype “Normal metabolizer” will depend on the number of variants investigated. The more variants analyzed (and found absent), the stronger the prediction “normal metabolizer” will be. Although there is a substantial agreement on this, each laboratory may have its own additional variants analyzed, usually depending on the genotyping platform used. It is therefore important that each laboratory also reports which SNPs were investigated. Clinical use of pharmacogenetics may benefit from consensus as to which variants should minimally be investigated. In 2018, the American Molecular Pathology (AMP) published a guideline for *CYP2C19* testing, giving as Tier 1 CYP2C19*2, *3, and *17 variant alleles and as Tier 2 CYP2C19*4A-*4B, *5, *6, *7, *8, *9, *10 and *35 alleles (Pratt et al., 2018). Tier 1 variant alleles were defined as those having: (i) well-characterized alteration of CYP2C19 activity that has been shown to have an effect on drug response and for which the functional variant is known, (ii) appreciable minor allele frequencies in a patient population, and (iii) available reference materials. Tier 2 alleles were defined as alleles that meet at least one, but not all of the criteria for inclusion in Tier 1 and are considered optional for expanded clinical genotyping panels. These include normal function variant alleles, low frequency alleles and alleles without available reference materials. In their recommendations, the differences in allele frequencies in different populations are taken into account. A similar initiative from AMP is currently ongoing for *CYP2D6* genotyping, but has not yet been published. In a recent article by Bousman C. et al. (2019), it was suggested that, in addition to *CYP2C19* and *CYP2D6*, *CYP2C9* (for phenytoin) and *HLA-A/HLA-B* gene variants should be considered for a ‘minimum, evidence-based genetic testing panel’ (Bousman C. et al., 2019).

Important is the conversion of SNPs into variant alleles using star allele assignments, with *1 being a default value, encoding active enzyme. There are currently 131 variant *CYP2D6* alleles described, which can be divided into active, decreased activity and inactive variants (Nofziger et al., 2020). Although polymorphisms affecting mRNA or protein expression will be following such a general categorization, it is important to keep in mind that particular variants causing amino acid substitutions may also cause changes in enzyme activity that are substrate dependent. A way to fine tune predicted phenotypes is an activity score (AS) assignment, with values 0 for non-functional alleles, 0.25, 0.5, and 0.75 for decreased activity alleles to 1.0 for active alleles (Gaedigk et al., 2008). The total score will indicate whether an individual is poor metabolizer (AS = 0), intermediate metabolizer (AS = 0.25–1.25), normal metabolizer (AS = 1.5–2.25), or ultra-rapid metabolizer (AS > 2.25). The challenge here is whether this conversion to PM, IM, NM, and UM should be maintained, as it lowers the information grade. Yet, clinicians may be accustomed to working with these phenotyping groups. Therefore, it remains to be seen whether the AS system will be adopted in routine clinical practice. In addition, one need to consider that most variants are detected by SNP analysis, and these analyses focus on the frequent variants described in literature. Next generation sequencing (NGS) would be helpful is analyzing CYP alleles in detail, also detecting not yet described variants. However, especially the *CYP2D6* locus seems to be complex to analyze, partly because of high homology with *CYP2D7* and *CYP2D8* pseudogenes. Lauschke and Ingelman-Sundberg (2019) reported on the value of NGS, now also successfully used for *CYP2D6*, stressing the value of rare variants. Of course this poses another challenge, as to assign a clinical relevance to rare variants identified that have not previously been characterized. Recent evidence suggests that bioinformatic tools may successfully be applied to NGS (Fabbri et al., 2020a). Specific programs that can be used for this are Aldy, Astrolable, and Stargazer. The value of NGS is that also rare variants can be detected. A drawback, however, can be that in a clinical setting, a variant cannot be assigned to a specific predicted phenotype, complicating the actions from a prescriber point of view. Another challenge for *CYP2D6* is the occurrence of gene deletions, multiplications, and *CYP2D6/7* hybrid alleles, excellently documented in a recent PharmVar review on *CYP2D6* (Nofziger et al., 2020). Copy number variation in CYP2D6 can be investigated by using CNV assays investigating signal strength at exon 9 or by analysis of specific PCR products, as done by XL-PCR (i.e., Autogenomics or Luminex). Approaches using two probes for CNV, like for intron 2 and exon 9, or the VeriDose approach (Agena BioSciences) utilizing 13 *CYP2D6* probes can be useful to get detailed information on the existence of hybrid alleles. The technical complexities of *CYP2D6* genotyping highlight the need for harmonization.

## Future Directions

It is challenging to harmonize the genotyping, since different platforms are in use, but it is clear that methods should be used that at least includes the AMP Tier 1 and 2 alleles that can detect hybrid alleles and that is FDA/CE-IVD approved. This then combined with clinical decision support software for conversion of genotyping to a specific dosing advice to help clinicians to better target their therapies. Of course, pharmacogenetics can be expanded from *CYP2D6* and *CYP2C19* genotypes to other genes, encoding enzymes, receptors, drug transporters, or other downstream molecules. From that point of view, there is still a lot to be discovered, with the challenge to see which genes do significantly improve therapeutic outcome. Implementation of *CYP2D6/CYP2C19* genotyping in psychiatry constitutes, in our opinion, an important first step in this.

## Conclusion

Analyzing today’s progress in clinical use of pharmacogenetics, we identify expert agreement on many aspects, but also still differences in opinion. As indicated, this concerns genotyping itself (SNP analysis/arrays/NGS), structural variant detection (haplotypes/CNVs/hybrids), genotype-to-phenotype translation, cost-effectiveness, and actionability (FDA/CPIC/PharmGKB lists). Notably, this paper did not discuss pharmacodynamic gene variants, since the clinical relevance is still under investigation (Fabbri et al., 2020b). Despite the challenges described, there is an increase in uptake for clinical care, making harmonization and clinical guidelines important to bring this field further in facilitating effective and safe treatment of patients.

## Author Contributions

All authors contributed to the article and approved the submitted version.

## Conflict of Interest

The authors declare that the research was conducted in the absence of any commercial or financial relationships that could be construed as a potential conflict of interest.

## Footnotes

1
www.pharmgkb.org – Clinical annotations (accessed June 1, 2020)

2
www.pharmgkb.org – Clinical annotations (accessed June 1, 2020)

3
https//cpicpgx.org (accessed June 1, 2020)

4
www.fda.org – Table of Pharmacogenomic Biomarkers in Drug Labelling (accessed June 1, 2020)

5
https://www.fda.gov/medical-devices/precision-medicine/table-pharmacogenetic-associations

6
https://www.fda.gov/medical-devices/safety-communications/fda-warns-against-use-many-genetic-tests-unapproved-claims-predict-patient-response-specific (accessed June 1, 2020)

7
www.upgx.eu
