# Supplementary material for: Pharmacogenetics in Psychiatry: An Update on Clinical Usability
Source: Front Pharmacol. 2020 Sep 11;11:575540. doi: 10.3389/fphar.2020.575540 (PMC7518035; doi:10.3389/fphar.2020.575540)
Supplement: Supplementary file 1 [file Table_1.pdf]

TABLE 1. Comparison of information on the FDA table of pharmacogenetic associations focusing on antidepressants and antipsychotics, compared with the DPWG and CPIC recommendations for antidepressant and antipsychotic drugs.

CYP2C19	FDA	PharmGKB – drug label annotation	PharmGKB – Clinical annotation		DPWG	CPIC
Amitriptyline	-	-	Level 1A	Efficacy	PK effects, no action	PM, RM and UM: alternative drug; IM: no action **
Citalopram	Therapeutic	Actionable (FDA/Can/Swiss)	Level 1A	Efficay/Toxicity	PM 50%; IM 75%; UM PK effects, no action	PM 50%; RM, UM: consider alternative drug
Clomipramine	-	-	Level 2A	Efficacy	PM and IM: PK effects, no action; UM: alternative drug	PM, RM, UM: alternative drug **
Doxepin	Pharmacokinetic	Actionable (FDA)	Level 3	Other	PK effects, no action	PM, RM, UM: alternative drug **
Escitalopram	Pharmacokinetic	Actionable (FDA/Japan/Swiss)	Level 1A	Efficay/Toxicity	PM 50%; IM 75%; UM alternative drug	PM 50%; RM, UM: alternative drug
Imipramine	-	-	Level 2A	Dosage / Other	PM 70%; IM and UM: PK effects, no action	PM 50%; RM and UM: alternative drug **
Moclobemide	-	Actionable (Swiss)	-	-	PK effects, no action	-
Sertraline	-	-	Level 1A	Metabolism/PK	PM max 75 mg/day; IM and UM: PK effects, no action	PM 50%
Trimipramine	-	Actionable (FDA)	Level 2A	Metabolism/PK	-	PM 50%; RM, UM: alternative drug **

CYP2D6	FDA	PharmGKB – drug label annotation	PharmGKB – Clinical annotation		DPWG	CPIC
Amitriptyline	Pharmacokinetic	Actionable (FDA)	Level 1A	Dosage/Efficacy	PM 70%; IM 75%; UM 140%	PM and UM: alternative drug; IM 75%
Atomoxetine	Pharmacokinetic	Actionable (FDA/Japan/Can/Swiss)	Level 1A	Efficacy/Toxicity	-	-
Aripiprazole	Therapeutic	Actionable (FDA/EMA/Can/Swiss)	Level 3	Other	PM max 10 mg/day; IM and UM: PK effects, no action	-
Brexpiprazole	Therapeutic	Actionable (FDA/EMA/Swiss)	-	-	PM 50%; IM and UM: PK effects, no action	-
Citalopram	-	Actionable (FDA/Swiss)	Level 3	Efficacy/Toxicity	No effect	-
Clomipramine	Pharmacokinetic	Actionable (FDA/Swiss)	Level 1A	Dosage/Toxicity/Efficacy	PM 50%; IM 70%; UM 150%	PM, UM: alternative drug; IM 75% **
Clozapine	Therapeutic	Actionable PGx (FDA)	-	-	No effect	-
Desipramine	Pharmacokinetic	Actionable PGx (FDA)	Level 1A	Dosage/Toxicity	-	PM, UM: alternative drug; IM 75%
Doxepin	Pharmacokinetic	Actionable PGx (FDA)	Level 1A	Dosage/Toxicity/Efficacy	PM 40%; IM 80%; UM 200%	PM, UM: alternative drug; IM 75% **
Duloxetine	-	Actionable (FDA/EMA/Swiss)	-	-	PK effects, no action	-
Escitalopram	-	Actionable (FDA/Japan)	Level 3	Dosage/Toxicity/Efficacy	No effect	-
Flupenthixol	-	-	-	-	No effect	-
Fluvoxamine	Pharmacokinetic	Actionable (FDA/Swiss)	Level 1A	Efficay/Toxicity	PM, IM and UM: PK effects, no action	PM 50-75%
Fluoxetine	-				PM, IM and UM: PK effects, no action	-
Haloperidol	-				PM 50%; IM PK effect, no action; UM: alternative drug	-
Imipramine	Pharmacokinetic	Actionable PGx (FDA)	Level 1A	Dosage/Toxicity	PM 30%; IM 70%; UM 170%	PM 50%; IM 75%; UM: alternative drug **
Iloperidone	Therapeutic	Actionable PGx (FDA)	Level 3	Toxicity/ADR	-	-
Mirtazapine	-	-	Level 2A	Efficay/Toxicity	PM, IM and UM: PK effects, no action	-
Nortriptyline	Pharmacokinetic	Actionable PGx (FDA/Can)	Level 1A	Dosage/Toxicity/Efficacy	PM 40%; IM 60%; UM 170%	PM 50%; IM 75%; UM: alternative drug
Olanzapine	-	Actionable PGx (FDA) / Informative (EMA)			No effect	-
Paroxetine	Pharmacokinetic	Informative (FDA)	Level 1A	Efficacy/Toxicity	PM and IM: PK effects, no action; UM alternative drug	-
Perphenazine	Safety/response	Actionable (FDA/Japan)	-	-	-	-
Pimozide	Therapeutic	**Testing required (FDA)**	Level 4	Other	PM max 10 mg/day; IM max 16 mg/day; UM PK effects, no action	-
Quetiapine	-	-	Level 4	Toxicity/ADR	No effect	-
Protriptyline	Pharmacokinetic	Actionable PGx (FDA)	-	-		-
Risperidon	Pharmacokinetic	Informative (FDA/Can/Swiss)	Level 2A	Efficay/Toxicity/Other	PM 50-67%; IM PK effects, no action; UM: alternative drug	-
Sertraline		-	Level 3	Dosage	-	
Tetrabenazide	Therapeutic	**ing required (FDA/Swiss) / Actionable (Japan/**	-	-	-	-
Thioridazide	Therapeutic	Actionable PGx (FDA)	Level 3	Other	-	-
Trimipramine	Pharmacokinetic	Actionable PGx (FDA)	Level 1A	Dosage/Toxicity/Other	-	PM 50%; IM 75%; UM: alternative drug **
Venlafaxine	Therapeutic	Actionable PGx (FDA/Swiss)	Level 2A	Efficay/Toxicity	PM and IM: PK effects, alternative drug; UM 150%	-
Zuclopenthixol	-	Informative (Swiss)	Level 3	Other	PM 50%; IM 75%; UM: alternative drug	-

*†) CYP2C19*1/*17 is defined as a rapid metabolizer (RM) in US. For DPWG, CYP2C19*1/*17 is regarded as a normal metabolizer (NM).*

IM = intermediate metabolizer, PM = poor metabolizer, UM = ultrarapid metabolizer.

***CPIC also has combined adviced based on both CYP2D6 and CYP2C19 genotype*

Note: sometimes both an alterantive dose as well as the option for an alternative drug is recommended. In that case, only the dose reduction is mentioned in this table
